# Supplementary material for: Using reflective practice to support PhD students in the biosciences
Source: eLife. 2024 Jan 23;13:e92365. doi: 10.7554/eLife.92365 (PMC10805382; doi:10.7554/eLife.92365)
Supplement: Supplementary file 1. [file elife-92365-supp1.docx]

**SoCoBio Wellbeing Survey April 2023**

***(This is given to students in a Microsoft Form and students can either select from a choice of answers or write in a blank box).***

**It's time for our annual wellbeing survey. We are interested in knowing how you are doing and would like to better understand what kind of support you need during your PhD, and how best to deliver it. This is particularly important given that we can provide help and support through the SoCoBio programme, as well as through your host Universities.

Responses are anonymous, and the survey takes less than 5 minutes to complete, so please give us your feedback to help us to improved the SoCoBio programme!

All the best
Dr Jenny Tullet
Wellbeing Lead for the SoCoBio DTP**

**1.Which DTP partner are you based at?**

University of Kent

NIAB at East Malling

University of Portsmouth

University of Southampton

University of Sussex

**2.Since October 2023, the beginning of this academic year, have you experienced issues that have affected your mental health/wellbeing**

Yes

No

Unsure

Not applicable

**3.Did these issues affect your ability to carry out your research/other activities associated with the Programme?**

Yes

No

Unsure

Not applicable

**4.Can you comment a bit further on what you think caused you to experience mental health/wellbeing issues? Leave blank if not applicable**

**5.How have you found your interaction with members of the Management Board and administration of the SoCoBio programme in supporting your wellbeing?**

Very helpful

Helpful

Unhelpful

very unhelpful

Not applicable

**6.How have you found your interaction with Postgraduate co-ordinators/Director of Doctoral Studies in your University around wellbeing issues?**

Very helpful

Helpful

Unhelpful

Very unhelpful

Not applicable

**7.If you needed to seek support for mental health issues, how easy did you find and utilse the support?**

Very easy

Easy

Difficult

Not applicable

**8.Which stress factors were the most important in causing any issues that you have experienced? Tick all that apply**

Lack of progress with project

Lack of support from others

High workload

Worries associated with physical or mental health

Social isolation

Uncertainty about the future

Personal or family circumstance

Conflict with supervisory team or others

Not applicable

**9.What do you think the SoCoBio DTP and /or your University could do to better support your mental health and wellbeing?**

In 2021 and 2022 an additional question was added between questions 3 and 4

**Q. If you have experienced issues that have affected your mental health and wellbeing, do you believe this was a result of the COVID-19 pandemic?**

Yes

No

Unsure

Not applicable
